# A rare and distinct hepatic tumor: Hepatocholangiocarcinoma

**DOI:** 10.1002/ccr3.3879

**Published:** 2021-02-02

**Authors:** Faten Limaiem, Saadia Bouraoui

**Affiliations:** ^1^ Tunis Faculty of Medicine University of Tunis El Manar Tunis Tunisia

**Keywords:** hepatocholangiocarcinoma, immunohistochemistry, liver, pathology, tumor

## Abstract

The rare histological features of hepatocholangiocarcinoma require a thorough sampling of the surgical specimen and the application of immunohistochemical techniques to confirm the diagnosis.

## Graphical abstract

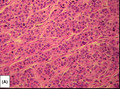

## 1 CLINICAL IMAGE

Hepatocholangiocarcinomas are rare primary malignant tumors of the liver (<5%) in which dual hepatocyte and biliary differentiation coexist.^1^clinicobiological and radiological characteristics are notclinicobiological and radiological characteristics are not Their clinicobiological and radiological characteristics are not very specific. Only the histopathological study coupled with the immunohistochemistry allows the diagnostic certainty.^2^

**FIGURE 1:**
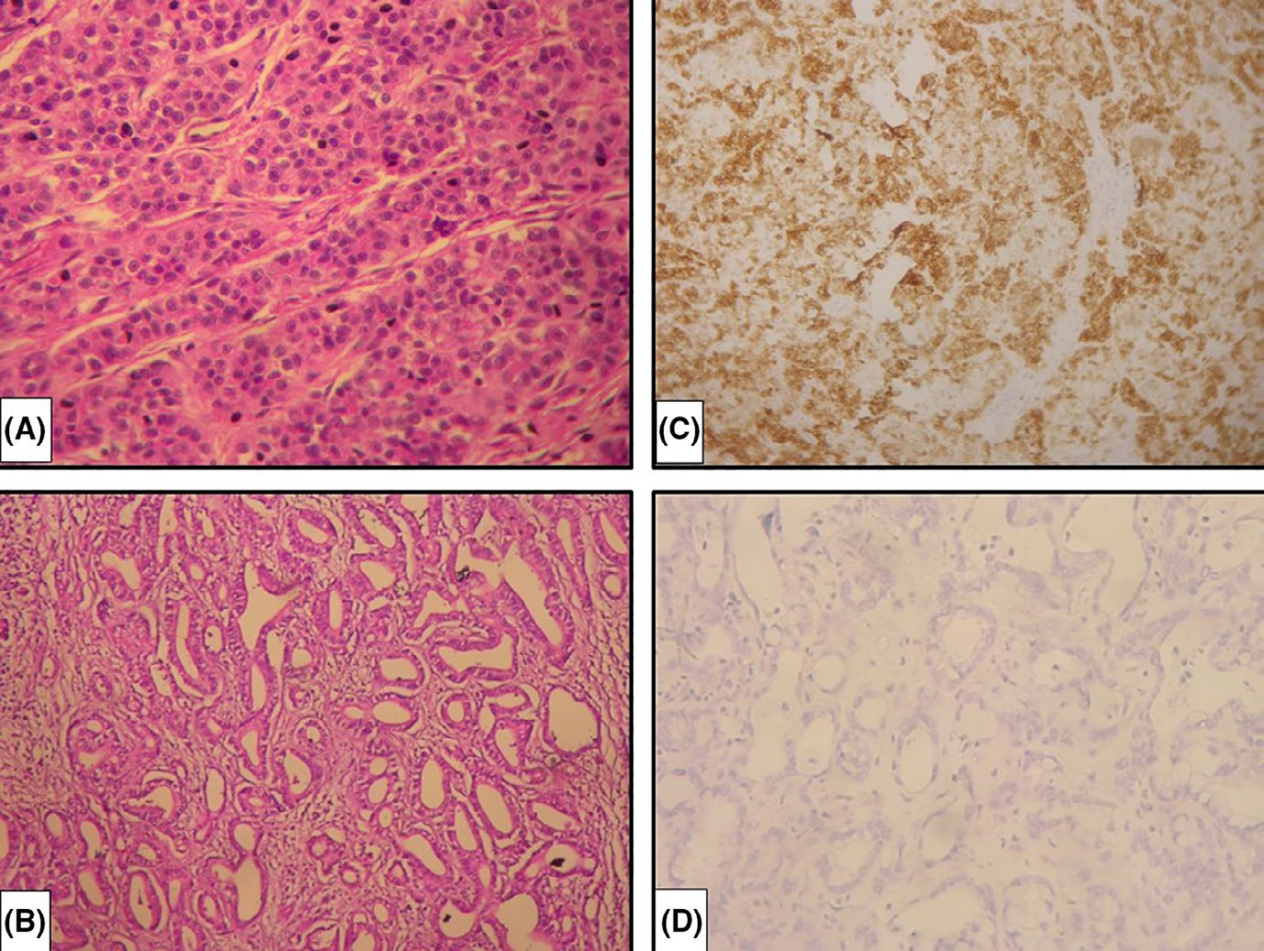
A, Tumor cells of the hepatoid sector organized in thick trabeculae (Hematoxylin and eosin, ×200). B, Tumor cells of the biliary component organized in glandular structures within a fibrous stroma (Hematoxylin and eosin, ×200). C, Hepatocyte‐type tumor cells expressed HepPar‐1 (Immunohistochemistry, ×200). D, The tumor cells of the biliary component were negative for HepPar‐1 (Immunohistochemistry, ×200)

A 49‐year‐old patient with no significant past medical history, underwent left hepatectomy for suspected hepatocellular carcinoma. Macroscopically, the surgical specimen measured 21 × 17 × 9 cm. On cut section, there was a firm yellowish‐white nodule with polycyclic contours measuring 13 × 12 × 8 cm. Histologically, the tumor consisted of two compartments of different appearance. One of the contingents was hepatoid with large cells, organized in thick, irregular trabeculae (Figure 1A). In other areas, the organization of tumor cells was glandular within a more abundant fibrous stroma (Figure 1B). Immunohistochemically, hepatocyte‐type tumor cells expressed HepPar‐1 (Figure 1C) and AFP but were negative for CK7 and CK19. In the second contingent of glandular architecture, the tumor cells expressed CK7 and CK19 but were negative for HepPar‐1 (Figure 1D). The final pathological diagnosis was that of hepatocholangiocarcinoma. The evolution was marked by tumor recurrence 2 years later requiring surgical reoperation.

## CONFLICT OF INTEREST

None declared.

## AUTHOR CONTRIBUTIONS

FL: prepared, organized, wrote, and edited all aspects of the manuscript. She performed the gross and microscopic pathologic evaluation of the pathology specimen. She prepared all of the histology figures in the manuscript. She read, edited, and approved the final version of the manuscript. SB: participated in the drafting of the article and revising it critically for important intellectual content.

## ETHICAL APPROVAL

All procedures performed were in accordance with the ethical standards. The examination was made in accordance with the approved principles.

## Data Availability

In accordance with the DFG Guidelines on the Handling of Research Data, we will make all data available upon request.
